# Antibacterial Potential of Secondary Metabolites from Indonesian Marine Bacterial Symbionts

**DOI:** 10.1155/2020/8898631

**Published:** 2020-06-29

**Authors:** Risa Nofiani, Alexandra J. Weisberg, Takeshi Tsunoda, Ruqiah Ganda Putri Panjaitan, Ridho Brilliantoro, Jeff H. Chang, Benjamin Philmus, Taifo Mahmud

**Affiliations:** ^1^Department of Chemistry, University of Tanjungpura, Pontianak 78124, Indonesia; ^2^Department of Pharmaceutical Sciences, Oregon State University, Corvallis, OR 97331, USA; ^3^Department of Botany and Plant Pathology, Oregon State University, Corvallis, OR 97331, USA; ^4^Department of Biological Education, University of Tanjungpura, Pontianak 78124, Indonesia

## Abstract

Indonesian marine environments are known to house diverse organisms. However, the potential for bacteria from these environments as a source of antibacterial agents has not been widely studied. This study aims to explore the antibacterial potential of secondary metabolites produced by bacterial symbionts from sponges and corals collected in the Indonesian waters. Extracts of 12 bacterial isolates from sponges or corals were prepared by cultivating the bacteria under a number of different media conditions and using agar well diffusion assays to test for antibacterial activity. In addition, the morphology, physiology, and biochemical characteristics and 16S rRNA sequence of each isolate were used to determine their taxonomic classification. All tested bacterial isolates were able to produce secondary metabolites with various levels of antibacterial activity depending on medium composition and culture conditions. Two of the bacteria (RS3 and RC4) showed strong antibacterial activities against both Gram-negative and Gram-positive bacteria. A number of isolates (RS1, RS3, and RC2) were co-cultured with mycolic acid-containing bacteria, *Mycobacterium smegmatis* or *Rhodococcus* sp. However, no improvements in their antibacterial activity were observed. All of the 12 bacteria tested were identified as *Streptomyces* spp. LC-MS analysis of EtOAc extracts from the most active strains RS3 and RC4 revealed the presence of a number of dactinomycin analogues and potentially new secondary metabolites. Symbiotic *Streptomyces* spp. from sponges and corals of the Indonesian marine environments have great potential as a source of broad-spectrum antibacterial agents.

## 1. Introduction

The widespread emergence of antibiotic-resistant pathogens has become a major healthcare problem worldwide. This problem is being compounded by the decline in the number of new antibiotics being discovered and developed in recent years [1, 2]. This has called for renewed efforts to find new antibiotics.

Actinobacteria have been known to be a rich source of bioactive compounds. Among the most productive genera of the Actinomycetales are *Streptomyces*, *Nocardiopsis*, and *Micromonospora*, members of which can be terrestrial or aquatic, including living symbiotically in various marine organisms such as sponges, corals, and seaweeds [3]. Bacteria associated with sponges have been reported to reach up to 40% of the sponge biomass [4, 5]. In coral, actinobacteria can be harbored in tissue and mucus with differences in abundance and diversity [6]. However, only around 0.001–1% of them are deemed to be culturable [7, 8]. Nevertheless, previous studies have shown that many sponge- and coral-associated bacteria produce more novel bioactive compounds than their counterparts that live in terrestrial environments [4, 9].

The Indonesian archipelago is among the places on the earth that house highly diverse marine organisms, including sponges and corals. However, their potential as a source of antibacterial agents has not been widely studied. Here, we report the antibacterial potential of secondary metabolites from a number of sponge- and coral-associated bacterial symbionts that were collected from Indonesian waters around West Kalimantan.

## 2. Materials and Methods

### 2.1. Sampling

Corals and sponges were collected in 2017 from waters around Baru Island, Randayan Island, and Lemukutan Island, Bengkayang District, West Kalimantan, Indonesia (Table 1). All samples were stored in the icebox and transferred to the laboratory.

### 2.2. Isolation of Coral- and Sponge-Associated Bacteria

The sponges and corals were rinsed with sterilized seawater, homogenized with a sterilized hand blender, and inoculated on agar plates containing ISP1 (International Streptomyces Project Medium 1, Difco) or ISP2 (International Streptomyces Project Medium 2, Difco) supplemented with nystatin (100 *μ*g/mL) and nalidixic acid (100 *μ*/mL) at room temperature (27°C–32°C). Spore-forming colonies growing on the agar were repeatedly transferred to new plates until pure isolates were obtained.

### 2.3. Production and Extraction

Seed cultures were prepared by inoculating bacterial spores into the ISP1 medium (50 mL) and grown at 30°C for 2–3 days with shaking at 200 RPM. The seed cultures were used for mono and co-culture experiments. Monoculture production was carried out in different media, e.g., modified Bennet with artificial seawater (Himedia) (MB + ASW), ISP1 with artificial seawater and trace element (ISP1 + ASW + TE), and amylostatin media (AM) [10–12]. The modified Bennet medium consists of glucose 10 g/L, yeast extract 1 g/L, beef extract 1 g/L, soytone 1 g/L, metal stock solution 1 mL, and pH adjusted to 7.35. The ASW composition is NaCl 24.6 g/L, KCl 0.67 g/L, CaCl_2_•2H_2_O 1.36 g/L, MgSO4•7H_2_O 6.29 g/L, MgCl_2_•6H_2_O 4.66 g/L, NaHCO_3_ 0.18 g/L, and pH adjusted to 7.5 ± 0.5. Fifty mL of the metal stock solution consists of FeSO_4_•7H_2_O 50 mg, CaCl_2_•2H_2_O 90 mg, MnCl_2_•4H_2_O 180 mg, CaCl_2_•6H_2_O 25 mg, CuSO_4_•5H_2_O 25 mg, ZnSO_4_•4H_2_O 25 mg, and (NH_4_)_6_Mo_7_O_24_•4H_2_O 25 mg. The amylostatin medium (AM) consists of maltose 5%, soy flour 2.5%, wheat germ 1%, NaCl 0.25%, and pH adjusted to 7. The trace element consists of ZnCl_2_ 40 mg/L, FeCl_3_ 6H_2_O 200 mg/L, CaCl_2_•2H_2_O 10 mg/L, MnCl_2_•4 H_2_O 10 mg/L, NaB_4_O_7_•10H_2_O 10 mg/L, and (NH_4_)_6_Mo_7_O_24_•4H_2_O 10 mg/L. A seed culture (5 mL) was inoculated into a 500 mL Erlenmeyer flask containing 100 mL of the medium and grown at 200 rpm, 30°C. After 7 days, the culture was harvested and centrifuged at 3,000 × *g* for 10 min, and the supernatant was extracted successively with ethyl acetate (EtOAc) and *n*-butanol (*n*-BuOH). A rotary evaporator was used to evaporate organic solvents to obtain EtOAc and *n*-BuOH extracts.

Co-culture experiments were carried out using isolates RS1, RS3, and RC2 and two counterpart bacteria, *Mycobacterium smegmatis* and *Rhodococcus* sp. The media used for these experiments were AM, BTT (glucose 3 g/L, yeast extract 3 g/L, Bactotryptone 5 g/L, and pH adjusted to 7.4) [13], and A3M [14]. Seed cultures of *M*. *smegmatis* and *Rhodococcus* sp. were prepared by inoculating the bacteria into ISP2 and Luria-Bertani (LB) media (tryptone 10 g/L, yeast extract 5 g/L, NaCl 10 g/L, and pH adjusted to 7), respectively, and grown at 200 rpm and 30°C for 3 days. Each seed culture (1 mL) was inoculated into the production media (50 mL) and grown at 200 rpm and 30°C for 7 days. The cultures were harvested and centrifuged, and the supernatants were extracted successively with EtOAc and *n*-BuOH. A rotary evaporator was used to evaporate organic solvents to obtain EtOAc and *n*-BuOH extracts.

### 2.4. Characterization of Bacterial Isolates

The physiological and biochemical characteristics of each isolate were examined based on spore color, diffusible pigment, melanoid pigmentation, salt tolerance, pH tolerance, hydrolysis of protein and starch, and utilization of carbohydrates [15]. The bacteria were grown on agar plates for 5–7 days at 30°C. Spore color, diffusible pigment, and melanoid pigmentation were evaluated on ISP1 and ISP2 agar. Salt and pH tolerances were evaluated on ISP2 agar. Hydrolysis of protein and starch was evaluated on the ISP2 medium enriched with milk (1%) and corn starch (1%), respectively. The utilization of carbohydrate was tested using basal mineral salt agar (BMSA) enriched with carbohydrates such as D-glucose (1%), D-galactose (1%), D-mannose (1%), D-arabinose (1%), D-xylose (1%), D-fructose (1%), maltose (1%), sucrose (1%), or inositol (1%) or dextrin (0.5%). The BMSA consists of (NH_4_)_2_SO_4_ 2.64 g/L, KH_2_PO_4_ anhydrous 2.38 g/L, K_2_HPO_4_•3H_2_O 5.65 g/L, MgSO_4_•7H_2_O 1 g/L, CuSO_4_•5H_2_O 0.0064 g/L, FeSO_4_•7H_2_O 0.0011 g/L, MnCl_2_•4H_2_O 0.0079 g/L, and ZnSO_4_•7H_2_O 0.0015 g/L and pH adjusted to 7.2–7.4 [16]. Each isolate was streaked with 1 cm in length on BMSA with different carbohydrate sources. Positive and negative controls for carbohydrate utilization were D-glucose and no carbohydrate source, respectively.

A large fragment of the 16S rRNA gene was sequenced to determine the taxonomic classifications for the isolates. The 16S rRNA gene was amplified using a pair of universal primers 27F (5′-AGAGTTTGGATCMTGGCTCAG-3′) and 1492R (5′-CGGTTACCTTGTTACGACTT-3′) and Takara Taq™ DNA polymerase. PCR was carried out according to the manufacturer's guidelines. The annealing temperature was set at 63°C. The PCR products were cleaned up using the EZNA purification kit and sequenced on an ABI3730 capillary sequencing machine at the Oregon State University Center for Genome Research and Biocomputing.

The EBI Clustal Omega webserver was used to calculate pairwise percent identity between all 16S rRNA gene sequences [17, 18]. BLAST searches were performed against the NCBI 16S rRNA gene database using 16S rRNA gene from isolates RS1 and RC4 as representative query sequences, and the top 500 and 132 hits were downloaded and combined for analysis. MAFFT v. 7.402 E-INS-i with the default parameters was used to align 16S rRNA gene sequences [19]. IQ-TREE v. 1.6.12 with the options “-bb 1000 -alrt 1000” was used to generate a phylogenetic tree with bootstrap support [20]. Phylogenies were midpoint-rooted and visualized using FigTree v1.4.4. All of the partial 16S rRNA gene sequences were submitted to GenBank with accession numbers MT011999 (RS1), MT012000 (RS5), MT012001 (RS6), MT012002 (RS7), MT012003 (RS2), MT012004 (RC2), MT012005 (RC5), MT012006 (RC3), MT012007 (RC4), and MT448898 (RS3).

### 2.5. Antibacterial Assay

*Staphylococcus aureus* ATCC 12600, *Bacillus subtilis* ATCC 6051*, Salmonella enterica* ATCC 14028*, Escherichia coli* ATCC 11775, *Pseudomonas aeruginosa* ATCC 9721, and *Mycobacterium smegmatis* ATCC 14468 were used as test bacteria. The test bacteria, except for *M*. *smegmatis*, were inoculated into the LB medium and grown overnight at 200 rpm, 37°C. *M*. *smegmatis* was inoculated into the ISP2 medium and grown at 200 RPM, 30°C for 3 days. Antibacterial assays were carried out using the agar well diffusion technique. Fifteen milliliters of warm LB agar was mixed with 300 *μ*L test bacterial culture and plated out in a petri dish with a diameter of 9 cm. After solidification, a sterilized puncher was used to punch a hole into the agar. The extract (20 *μ*L, 10 *μ*g/*μ*L) was then loaded into the well. Once the extract dried, the plate was then incubated at 37°C (30°C for *M*. *smegmatis*). Antibacterial activity was determined based on the formation of an inhibition zone and the diameter of the zone.

### 2.6. LC-MS Analysis of Strains RS3 and RC4

High-resolution mass spectrometry (HR-MS) was obtained using an Agilent 1260 HPLC upstream of an Agilent 6545 Q-ToF. The separation was achieved using InfinityLab Poroshell EC-C18 column (100 × 3.0 mm, 2.7 *μ*m) at a flow rate of 0.4 mL/min and the following gradient. Line A was water with 0.1% (v/v) formic acid, and line B was acetonitrile with 0.1% (v/v) formic acid. The column was preequilibrated with 90% A/10% B. Upon injection, the mobile phase composition was maintained for 1 minute upon when the mobile phase was changed using a linear gradient to 0% A/100% B over the following 20 minutes. This concentration was held for 6 minutes followed by changing the mobile phase to 90% A/10% B over 1 min. The Q-ToF mass spectrometer was operated in the Auto MS/MS mode.

The Q-ToF machine was operated using MassHunter software, and data were processed offline, using MassHunter Qualitative Analysis software. Compounds were identified for Global Natural Products Social Molecular Networking (GNPS) analysis using the “Compounds Discovery” workflow with the “Auto-Select Compound Mining” settings [21]. MS/MS peaks and spectrum were chosen according to the following settings: average scans, 10% of peak height; exclude, if above 10% of saturation; no MS/MS spectrum background; height filters, absolute height, 10 counts, relative height, 1% of largest peak; charge state isotope model, unbiased; limit-assigned charge states to a range of 1-2. The files were then exported as both MGF and MzData formats and used for molecular networking.

## 3. Results and Discussion

### 3.1. Origin of the Isolates

Two hundred and five bacterial isolates were cultured from unidentified sponges and corals collected at a number of locations around Baru, Lemukutan, and Randayan islands off the coast of West Kalimantan, Indonesia. Among them, 12 isolates which showed velvety or powdery colonies characteristic to *Streptomyces* were selected for further study (Table 1). In this study, streptomycetes were prioritized due to their complex secondary metabolism and their potential as a source of antibacterial agents. The five coral-associated isolates were designated as RC1–RC5, and the seven sponge-associated bacterial isolates were designated as RS1–RS7.

### 3.2. Characterization of the Bacterial Isolates

**Figure 1:**
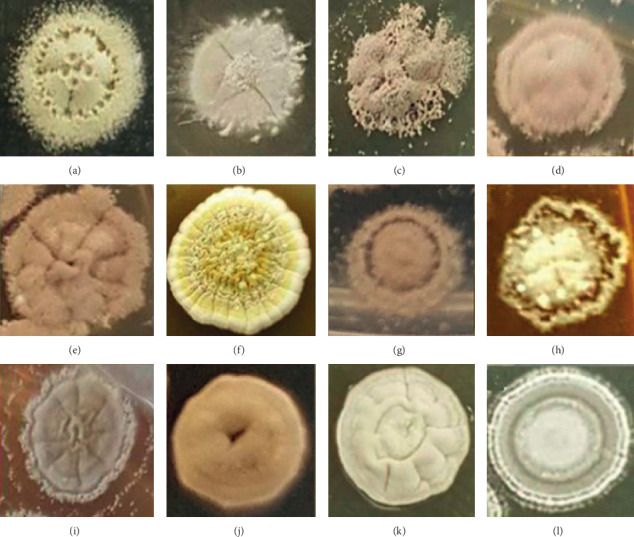
Morphology of each isolate colony grown on the ISP2 medium: (a) RC1, (b) RC2, (c) RC3, (d) RC4, (e) RC5, (f) RS1, (g) RS2, (h) RS3, (i) RS4, (j) RS5, (k) RS6, and (l) RS7.

Preliminary identification of actinobacteria is normally performed by observation of sporophore morphology and color, soluble pigment, and different growth rates on various media [22]. Each of the 12 isolates produced spores on ISP1 and ISP2 media (Table 2). On the ISP1 medium, all of them produced white spores, but when grown on the ISP2 medium, they exhibited different colors of spores, i.e., white, pale yellow, greenish, or grey (Figure 1). All spores have velvety or powdery form and pigmentation which are the main characteristics for *Streptomyces* [23]. Pigments produced by bacteria were grouped into two types: melanoid and other pigments [16]. Melanoid pigment production is indicated by a brown or black appearance on the reverse side of the colony. Bacteria may also produce other pigments such as red, yellow, green, blue, or violet, which are coded as distinctive (d), whereas pale yellow, olive, or yellowish brown are considered non-distinctive [22, 23]. None of the 12 isolates produced any pigments when growth on ISP1 agar (Table 2). However, five isolates (RS1, RS3, RS4, RS5, and RC4) produced melanoid pigment on ISP2.

None of the isolates grew at pH 4.0, few grew at pH 5.0, but all grew between pH 6.0 and pH 13.0 (Table 2). Therefore, these isolates fall into the group of alkaliphilic bacteria [24, 25]. All of the isolates grew on ISP1 containing 0–5% NaCl, although some were highly resistant to salinity and grew on ISP1 containing NaCl up to 12.5%. Based on these results, all of the isolates were categorized as halotolerant due to their ability to grow in the presence of NaCl [20, 26]. As a number of isolates grew optimally at pH 9.0 or above and in high salinity, they are categorized as haloalkaliphiles [25]. While all of the tested isolates were generally able to utilize D-glucose, D-galactose, or dextrin as the sole carbon source, many of them also utilized D-mannose, D-arabinose, D-xylose, D-fructose, maltose, inositol, and/or sucrose. Based on their Gram staining, morphology, spore formation ability, and physiological characteristics, all of the isolates were predicted to be actinomycetes (Table 2).

### 3.3. Genetic Analysis of the Bacteria

**Figure 2:**
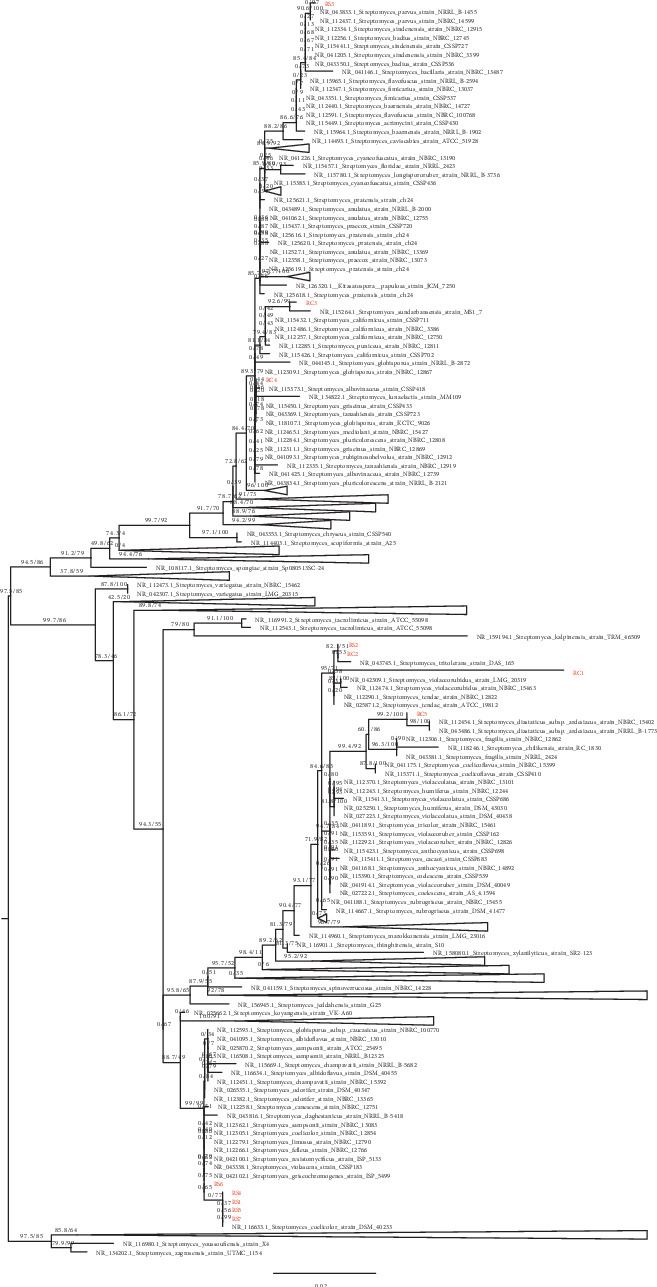
Phylogenetic tree of the 16S rRNA gene from each isolate and representative strain from NCBI GenBank. Labels of strains from this study are colored red. Branches are labelled with ultrafast bootstrap and SH-aLRT support values. The tree is midpoint rooted. Some clades are collapsed and are represented as triangles.

The isolates were also genetically examined by sequencing their 16S rRNA gene fragments, and the sequences were analyzed using BLASTN searches against the NCBI 16S database. The results indicate that all of the strains are *Streptomyces* spp. The 16S rRNA gene sequences were also compared with similar strains available in the GenBank database, and a phylogenetic tree was constructed to observe the evolutionary relationship between the isolates (Figure 2).

Subsequently, the pairwise identity between isolates was also calculated (Table 3). The phylogeny and pairwise comparisons revealed that many of these isolates are genetically distinct from each other and represent multiple *Streptomyces* lineages. Some isolates have 16S rRNA gene sequences similar to those of previously sequenced strains. The 16S rRNA gene sequences of the strains RC2 and RS2 are very similar to the 16S rRNA gene sequence of *Streptomyces tritolerans* strain DAS 165 (Figure 2). Others are more unique (RC1, RC5) and are less similar to previously identified strains. However, a number of new isolates were highly similar to each other in 16S gene sequence. The newly sequenced isolates RS1, RS5, RS6, and RS7 have identical 16S rRNA gene sequences, despite the fact that they were isolated from different sponges that were collected from different locations (Table 1). They also displayed different morphological and physiological characteristics, suggesting that they vary genetically despite sharing similar 16S rRNA gene (Figure 1 and Table 3). Similarly, isolate RS3, which was isolated from a sponge, has an identical 16S rRNA gene sequence with that of RC4, which was isolated from a coral.

### 3.4. Evaluation of Antibacterial Activity

To evaluate the potential of the isolates as a source of antibacterial compounds, the isolates were cultivated in a number of different media. As all of the isolates were able to grow in cultures with or without salt, three isolates (RS3, RS4, and RS5) were randomly selected and cultivated in the ISP1 medium with or without artificial salt water (ASW) to determine if ASW could influence antibacterial production. The culture broths were then extracted successively with EtOAc and *n*-BuOH, and the extracts were tested for their antibacterial activity. The results showed that extracts from the strains RS3 and RS5 cultivated in ISP1+ASW had stronger antibacterial activities than those produced from strains cultivated in ISP1 alone (Table 4). However, extracts of strain RS4 cultivated in ISP1+ASW did not show better antibacterial activities than those cultivated in the ISP1 medium alone.

Next, the effects of trace element (TE) supplementation on antibiotic production were evaluated. In this study, isolates RS3, RS4, and RS5 were cultivated in ISP1 + ASW + TE and the products were tested for their antibacterial activity. The results showed that the addition of TE negatively impacted the production of antibiotics in the RS3 and RS5 cultures, as extracts from RS3 and RS5 cultivated in ISP1 + ASW + TE had lower antibacterial activities than those in ISP1 + ASW. However, extracts from the ISP1 + ASW + TE culture of RS5 were more active than those without TE (Table 4).

Based on the above results, three different media (i.e., MB + ASW, ISP1 + ASW + TE, and AM) with very different carbon and nitrogen source compositions were selected for further studies. The primary carbon sources in MB and AM media are glucose and maltose, respectively, whereas in the ISP1 medium, the carbon sources are yeast extract and peptone. All of the media contain nitrogen sources such as tryptone and yeast extract (in the ISP1 medium), soy flour and wheat germ (in the AM medium), or yeast extract, beef extract, and soytone (in the MB medium). All of the isolates were cultivated in these media, and the extracts were tested against *S. aureus*, *B. subtilis*, *S. enterica*, *E. coli*, *P. aeruginosa*, and *M. smegmatis,* using an agar diffusion assay (Table 4). The results showed that all of the isolates produce antibacterial compounds in one or more of the media used. Most extracts are active against *S. aureus*, *B. subtilis*, *S. enterica*, and *E. coli*, whereas some of them are also active against *M. smegmatis*. Some of them (RC3, RC4, and RS3) showed growth inhibition zones equal to or better than the positive control tetracycline (2 *μ*g). Of particular interest was extracts from isolates RC3, RC4, RS3, and RS4, which were active against the highly difficult to treat Gram-negative pathogen *P. aeruginosa*.

### 3.5. Effects of a Co-culture on Antibacterial Production

Another strategy to stimulate the expression of biosynthetic gene clusters is by promoting interspecies interactions between microorganisms. Mycolic acid-containing bacteria, e.g., *Gordonia* sp., *Tsukamurella* sp., *Rhodococcus* sp., and *Mycobacterium* sp., have been shown to be good inducers of biosynthetic gene expression [14]. In the current study, we tested the effects of co-culture on antibiotic production of the marine bacterial symbionts using *Mycobacterium smegmatis* and/or *Rhodococcus* sp. as the counterpart. Isolates RS1, RS2, and RC2 were cultivated alone or together with *M. smegmatis* in AM, BTT, and/or A3M media. Isolate RS2 was also cultivated with *Rhodococcus* sp. The EtOAc and/or *n*-BuOH extracts of the culture broths were tested against *S. aureus*, *B. subtilis*, *E. coli*, *P. aeruginosa*, and *M. smegmatis*. However, no significant improvement of the antibacterial activity of the co-culture extracts was observed compared to those of the monocultures (Table 5). In some cases, the co-cultures negatively affected antibiotic production by the marine symbionts. It may be concluded that, under described experimental conditions, the mycolic acid-containing bacteria were not able to induce the production of antibacterial compounds in isolates RS1, RS2, and RC2; however, their ability to increase the production of other compounds in which no antibacterial activity has not been ruled out.

### 3.6. LC-MS Analysis of Strains RS3 and RC4

**Figure 3:**
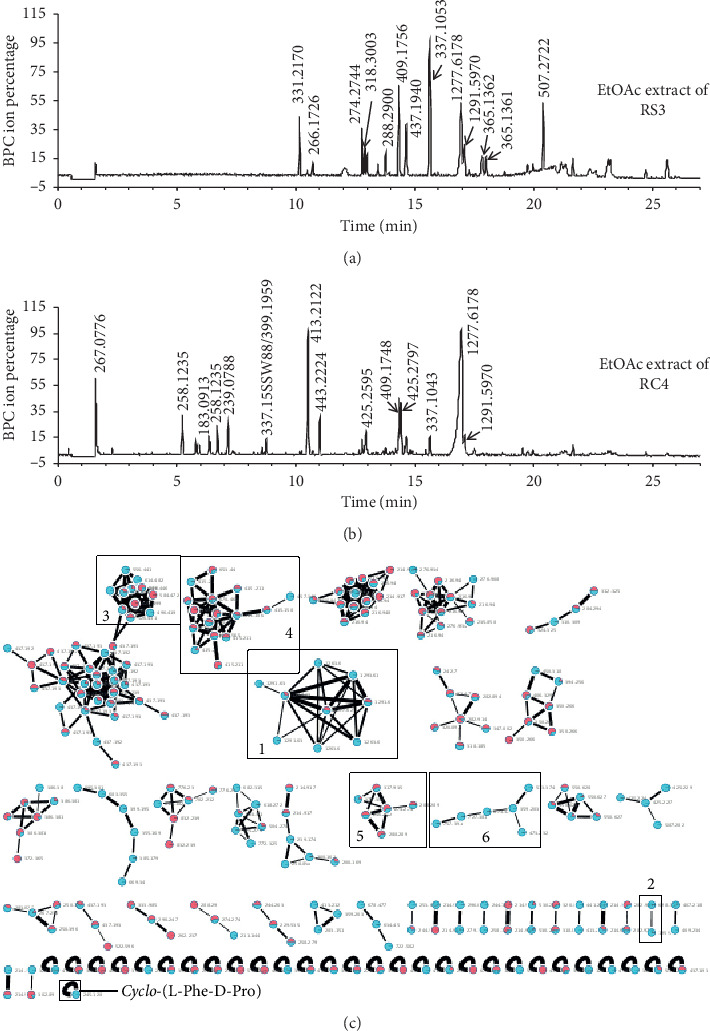
LC-MS analysis of EtOAc extracts from strains RS3 and RC4. (a) LC-MS chromatogram of EtOAc extract from strain RS3; (b) LC-MS chromatogram of EtOAc extract from strain RC4; (c) molecular networks of combined molecular ions of metabolites from strains RS3 and RC4. Red, strain RS3, and cyan, strain RC4. 1 and 2 are networks of the putative dactinomycin cluster (Na^+^ and H^+^ adducts, respectively); 3–6 are networks of unknown clusters. There is also a molecular ion for a putative *cyclo*-(L-Phe-D-Pro).

As strains RS3 and RC4 showed the greatest antibacterial activity (Table 4), we analyzed the extracts via HPLC-HRMS/MS followed by spectral networking using the GNPS platform [21]. The GNPS program identified a spectral match for one of our compounds with actinomycin D (dactinomycin) as well as *cyclo*-(L-Phe-D-Pro) (Figure 3). In both RS3 and RC4, we observed a compound displaying a sodiated ion of 1277.6178 which is consistent with actinomycin D (1277.6183, 0.4 ppm error). The GNPS algorithm detected related peaks at 1291.5970, 1263.5989, and 1293.6099 in RC4 and 1291.5970 in RS3 extracts. The sodiated ion at 1291.5970 is consistent with actinomycin Y5, actinomycin Z4, or actinomycin Y6 while the sodiated ions at 1263.5989 and 1293.6099 represent previously undescribed actinomycin derivatives lacking a methyl group (either an *N*-methyl, or substituting Thr for Ser) or with an oxidized variant (“+O”), respectively. We note that the compound generating the protonated ion at 1277.6178 is a prominent peak in both extracts and reasonably explains the observed antibacterial activity of the extracts. We also note other clusters of compounds unannotated by GNPS (Figure 3(c), boxes 3–6), which demonstrates the potential for our collection of Indonesian marine bacterial symbionts to yield new bioactive compounds.

## 4. Conclusion

From 205 bacterial isolates obtained from unidentified sponges and corals collected off the coast of West Kalimantan, Indonesia, 12 strains have been identified to be members of the *Streptomyces* spp. All 12 of the strains showed the ability to produce potent antibacterial compounds, and some of them were active against the opportunistic Gram-negative bacterial pathogen *P. aeruginosa*. The ability of the strains to produce antibacterial compounds is greatly dependent on the production medium and culture condition. Interestingly, co-cultures of selected strains with mycolic acid-containing bacteria did not improve the production of antibacterial compounds in those strains. Metabolic profiling of extracts from the most active strains indicates the presence of known antibacterial agents (the actinomycins and a *cyclo*-(L-Phe-D-Pro)), as well as a number of unidentified natural products. The results underscore the great potential of marine bacterial symbionts as a highly promising source of new antibiotics.

## Figures and Tables

**Table 1:** Bacterial isolates used in this study.

Isolate name	Source	Location	Notes
RC1	Coral	Baru Island, West Kalimantan	7-meter depth, 0°36′23.42″U : 108°45′22.41″T
RC2	Coral	Baru Island, West Kalimantan	7-meter depth, 0°36′23.42″U : 108°45′22.41″T
RC3	Coral	Baru Island, West Kalimantan	7-meter depth, 0°36′23.42″U : 108°45′22.41″T
RC4	Coral	Baru Island, West Kalimantan	7-meter depth, 0°36′23.42″U : 108°45′22.41″T
RC5	Coral	Baru Island, West Kalimantan	7-meter depth, 0°36′23.42″U : 108°45′22.41″T
RS1	Sponge	Lemukutan Island, West Kalimantan	n/a
RS2	Sponge	Randayan island, West Kalimantan	n/a
RS3	Sponge	Baru Island, West Kalimantan	7-meter depth, 0°36′23.42″U : 108°45′22.41″T
RS4	Sponge	Baru Island, West Kalimantan	7-meter depth, 0°36′23.44″U : 108°45′22.41″T
RS5	Sponge	Baru Island, West Kalimantan	7-meter depth, 0°36′23.42″U : 108°45′22.41″T
RS6	Sponge	Baru Island, West Kalimantan	7-meter depth, 0°36′23.42″U : 108°45′22.41″T
RS7	Sponge	Baru Island, West Kalimantan	7-meter depth, 0°36′23.42″U : 108°45′22.41″T

**Table 2:** Morphological and biochemical characteristics of the marine bacteria isolates RC1–RC5 and RS1–RS7.

Characteristics	Isolate											
	RC1	RC2	RC3	RC4	RC5	RS1	RS2	RS3	RS4	RS5	RS6	RS7
Gram staining	+	+	+	+	+	+	+	+	+	+	+	+
Spore color												
ISP1	White	White	White	White	White	White	White	White	White	White	White	White
ISP2	Grey	Grey	Pale yellow	Pale yellow	Grey	Olive	Grey	White	Greenish	Pale yellow	White	White
Pigment												
ISP1	nd	nd	nd	d	nd	nd	nd	nd	nd	nd	nd	nd
ISP2	d	nd	nd	d	nd	d	nd	d	d	d	d	nd
Melanoid pigmentation												
ISP1	—	—	—	—	—	—	—	—	—	—	—	—
ISP2	—	—	—	Brown	—	Black	—	Orange	Brown	Brown	—	—
NaCl tolerance												
0% NaCl	+	+	+	+	+	+	+	+	+	+	+	+
5% (w/v) NaCl	+	+	+	+	+	+	+	+	+	+	+	+
7.5% (w/v) NaCl	+	+	+	+	+	+	+	+	+	−	−	±
10% (w/v) NaCl	+	+	±	+	+	+	+	−	+	−	−	−
12.5% (w/v) NaCl	+	+	−	−	−	+	−	−	+	−	−	−
15% (w/v) NaCl	−	−	−	−	−	−	−	−	−	−	−	−
17.5% (w/v) NaCl	−	−	−	−	−	−	−	−	−	−	−	−
pH tolerance												
pH 4.0	−	−	−	−	−	−	−	−	−	−	−	−
pH 5.0	+	+	−	−	+	−	+	−	−	−	−	−
pH 8.0	+	+	+	+	+	+	+	+	+	+	+	+
pH 9.0	+	+	+	+	+	+	+	+	+	+	+	+
pH 10.0	+	+	+	+	+	+	+	+	+	+	+	+
pH 11.0	+	+	+	+	+	+	+	+	+	+	+	+
pH 12.0	+	+	+	+	+	+	+	+	+	+	+	+
pH 13.0	+	+	+	+	+	+	+	+	+	+	+	−
pH 13.80	−	−	−	−	−	−	−	−	−	−	−	−
Hydrolysis of												
Protein	+	+	−	−	−	+	+	+	+	−	−	−
Starch	+	+	−	+	+	+	−	+	+	+	+	+
Utilization of carbohydrate												
D-glucose	+	+	++	++	++	+	++	++	++	++	+	++
D-galactose	++	++	+	++	++	++	++	++	++	++	++	++
D-mannose	−	−	−	++	−	++	+	++	++	++	++	++
D-arabinose	++	++	−	++	++	++	++	+	++	++	+	+
D-xylose	+	++	−	+	+	++	++	++	++	++	++	+
D-fructose	++	++	++	++	++	++	++	++	++	++	++	+
Maltose	−	−	−	++	−	+	−	++	+	+	+	−
Dextrin	+	+	++	++	++	++	++	++	++	++	++	++
Inositol	++	++	−	+	+	+	++	+	+	+	++	−
Sucrose	−	−	−	−	+	+	+	+	+	+	+	−

*Note.* Sign for pigment tests: d, distinctive; nd, not distinctive. Sign for melanoid pigment: —, no melanoid pigment; sign for salt and pH tolerance: +, growth; −, no growth; sign for the carbohydrate utilization: ++, the isolate growth on tested carbon in the basal medium is equal to or greater than growth on the basal medium plus glucose; +, when the isolate growth on tested carbon in the basal medium was significantly better than that in the basal medium without carbon, but somewhat less than on glucose; —, when the isolate growth was similar to or less than growth in the basal medium without carbon.

**Table 3:** The matrix of pairwise identity of the 16S rRNA gene of each isolate.

	RC1	RS4	RC3	RS3	RC4	RS1	RS5	RS6	RS7	RS2	RC2	RC5
RC1	100	88.56	90.92	89.78	91.72	92.98	91.78	91.82	91.82	94.07	94.06	93.61
RS4	88.56	100	90.71	91.06	91.5	95.45	95.46	95.46	95.46	93.11	93.11	92.12
RC3	90.92	90.71	100	99.52	99.83	95.14	95.14	95.14	95.14	95.86	95.86	95.12
RS3	89.78	91.06	99.52	100	100	95.25	95.15	95.19	94.9	95.73	95.74	94.9
RC4	91.72	91.5	99.83	100	100	96.29	96.29	96.29	96.29	97.15	97.15	96.48
RS1	92.98	95.45	95.14	95.25	96.29	100	100	100	100	97.74	97.74	97.13
RS5	91.78	95.46	95.14	95.15	96.29	100	100	100	100	97.76	97.76	97
RS6	91.82	95.46	95.14	95.19	96.29	100	100	100	99.71	97.78	97.76	97.01
RS7	91.82	95.46	95.14	94.9	96.29	100	100	99.71	100	97.48	97.76	97.01
RS2	94.07	93.11	95.86	95.73	97.15	97.74	97.76	97.78	97.48	100	100	98.46
RC2	94.06	93.11	95.86	95.74	97.15	97.74	97.76	97.76	97.76	100	100	98.46
RC5	93.61	92.12	95.12	94.9	96.48	97.13	97	97.01	97.01	98.46	98.46	100

**Table 4:** Antibacterial activities of EtOAc extracts prepared with different media.

Isolate	Medium	Dose, *μ*g/well	Diameter of inhibition zone, mm					
			SA	BS	SE	EC	PA	MS
RC1	MB + ASW	200	—	—	—	—	—	—
	ISP1 + ASW + TE	200	8	10	10	8	—	—
	AM	200	10	14	14	10	—	4

RC2	MB + ASW	200	8	8	6	10	—	4
	ISP1 + ASW + TE	200	12	14	14	12	—	4
	AM	200	—	4	2	—	—	2

RC3	MB + ASW	200	12	20	14	14	—	6
	ISP1 + ASW + TE	200	12	18	18	12	—	14
	AM	200	16	22	14	10	4	16

RC4	MB + ASW	200	20	22	22	20	8	20
	ISP1 + ASW + TE	200	24	24	24	30	4	10
	AM	200	30	24	30	30	4	12

RC5	MB + ASW	200	—	—	8	—	—	2
	ISP1 + ASW + TE	200	—	—	—	—	—	—
	AM	200	10	10	8	10	—	—

RS1	MB + ASW	200	2	2	2	1	—	3
	ISP1 + ASW + TE	200	6	6	6	2	—	—
	AM	200	8	—	16	—		—

RS2	MB + ASW	200	3	18	2	1	—	6
	ISP1 + ASW + TE	200	6	8	9	4	—	—
	AM	200	4	8	4	4	2	4

RS3	MB + ASW	200	22	20	22	24	4	4
	ISP1	200	14	12	16	10	—	8
	ISP1 + ASW	200	20	20	21	20	6	14
	ISP1 + ASW + TE	200	10	10	8	12	—	1
	AM	200	2	16	22	24	1	—

RS4	MB + ASW	200	11	18	16	11	—	8
	ISP1	200	—	—	2	—	—	1
	ISP1 + ASW	200	2	—	—	—	—	—
	ISP 1ASW + TE	200	6	12	12	8	—	—
	AM	200	1	4	3	2	—	4

RS5	MB + ASW	200	12	1	12	12	—	12
	ISP1	200	2	1	—	1	—	—
	ISP1 + ASW	200	6	6	8	6	—	—
	ISP1 + ASW + TE	200	—	—	—	—	—	—
	AM	200	4	4	6	3	—	3

RS6	MB + ASW	200	4	4	4	4	—	2
	ISP1 + ASW + TE	200	—	1	—	—	—	—
	AM	200	8	12	—	—	—	2

RS7	MB ASW	200	5	6	2	—	—	—
	ISP1 + ASW + TE	200	8	12	—	—	—	6
	AM	200	—	—	2	—	—	—

Tetracycline (+control)		2	22	20	20	22	ND	ND

Rifampicin (+control)		8	ND	ND	ND	ND	ND	0.4

EtOAc (– control)		20 *μ*L	—	—	—	—	—	—

—, no inhibition zone; MB, modified Bennet; ASW, artificial seawater; TE, trace elements; AM, amylostatin medium; SA, *Staphylococcus aureus*; BS, *Bacillus subtilis*; SE, *Salmonella enterica*; EC, *Escherichia coli*; PA, *Pseudomonas aeruginosa*; MS, *Mycobacterium smegmatis*; ND, not determined.

**Table 5:** Antibacterial activities of co-culture extracts.

Isolate 1	Isolate 2	Medium	Extract	*μ*g/well	Antibacterial activities				
					SA	BS	EC	PA	MS
RS1	—	AM	EtOAc	200	++	−	−	−	−
RS1	*M*. *smegmatis*	AM	EtOAc	200	+	−	−	−	−
RS1	—	A3M	EtOAc	200	−	−	−	−	−
RS1	*M*. *smegmatis*	A3M	EtOAc	200	−	−	−	−	+
RS2	—	AM	EtOAc	200	++	+	++	+	++
RS2	*M*. *smegmatis*	AM	EtOAc	200	+	++	+	+	++
RS2	—	AM	*n*–BuOH	200	+	−	−	−	−
RS2	*M*. *smegmatis*	AM	*n*–BuOH	200	+	−	−	−	−
RS2	—	BTT	EtOAc	200	++	+	+	++	ND
RS2	*M*. *smegmatis*	BTT	EtOAc	200	++	++	+	++	ND
RS2	—	AM	EtOAc	200	+	−	−	+	+
RS2	*Rhodococcus* sp.	AM	EtOAc	200	+	−	−	−	+
RC2	—	AM	EtOAc	200	+	−	−	−	+
RC2	*M*. *smegmatis*	AM	EtOAc	200	+	−	−	−	+
RC2	—	AM	*n*–BuOH	200	+	−	−	−	−
RC2	*M*. *smegmatis*	AM	*n*–BuOH	200	+	−	−	−	+
RC2	—	BTT	EtOAc	200	−	−	−	−	ND
RC2	*M*. *smegmatis*	BTT	EtOAc	200	−	−	−	−	ND
RC2	—	BTT	*n*–BuOH	200	+	−	−	−	ND
RC2	*M*. *smegmatis*	BTT	*n*–BuOH	200	+	−	−	−	ND
RC2	—	A3M	EtOAc	500	−	−	−	−	−
RC2	*M. smegmatis*	A3M	EtOAc	500	−	−	−	−	+

+, activity showing inhibition zone; −, no activity showing no inhibition zone; ND, not determined; EtOAc, ethyl acetate; n-BuOH, n-butanol; SA, *Staphylococcus aureus*; BS, *Bacillus subtilis*; EC, *Escherichia coli*; PA, *Pseudomonas aeruginosa*; MS, *Mycobacterium smegmatis*.

## Data Availability

Data are available from GenBank to which all of the partial 16S rRNA sequences were submitted with accession numbers MT011999 (RS1), MT012000 (RS5), MT012001 (RS6), MT012002 (RS7), MT012003 (RS2), MT012004 (RC2), MT012005 (RC5), MT012006 (RC3), MT012007 (RC4), and MT448898 (RS3).
